# Psychological Stress Triggers a Hyperammonemia Episode in Patient with Ornithine Transcarbamylase Deficiency

**DOI:** 10.3390/ijerph191811516

**Published:** 2022-09-13

**Authors:** Valentín Emilio Fernández-Elías, José Francisco Tornero-Aguilera, Jose Alberto Parraca, Vicente Javier Clemente-Suárez

**Affiliations:** 1 Faculty of Sport Sciences, Universidad Europea de Madrid, Villaviciosa de Odón, 28670 Madrid, Spain; 2 Departamento de Desporto e Saúde, Escola de Saúde e Desenvolvimento Humano, Universidade de Évora, Largo dos Colegiais, 7000 Évora, Portugal; 3 Comprehensive Health Research Centre (CHRC), Universidade de Évora, 7000 Évora, Portugal

**Keywords:** psychological stress, hyperammonemia, case report, Ornithine Transcarbamylase deficiency (OTCD), metabolic stress response

## Abstract

An 18-year-old male motorcycle racer, who was a participant in the FIM Road Racing World Championship and had a history of Ornithine Transcarbamylase deficiency, developed nausea and dizziness while driving his motorcycle and became unconscious right after he stopped at the box. He was rapidly attended to by the medical personnel of the circuit, and once he recovered consciousness, he was taken to the local hospital where the blood analysis showed hyperammonemia (307 μg/dL) and excess alkalosis. The patient was properly following the prescribed treatment, and there were no environmental stressors. Hence, psychological stress and its somatization due to the risky task that the patient was performing could have triggered the episode. Stress must be considered as a potential cause, triggering strenuous metabolic stress that leads to hyperammonemia.

## 1. Introduction

Ornithine Transcarbamylase (OTC) is an enzyme responsible for catalyzing the reaction between carbamoyl phosphate and ornithine in order to form citrulline and phosphate; it plays a critical role in the urea cycle, which prevents the accumulation of toxic levels of ammonia in the organism [1]. The urea cycle is the process by which the ammonia produced in the gut, kidneys, and muscles is transported to the bloodstream, carried to the liver, and transformed into urea for excretion [2]. OTC deficiency is caused by a mutation in the OTC gene (Xp21.1), which encodes the OTC. It is classified as a urea cycle disorder since without a correctly functioning OTC, ammonia levels increase in the blood and produce hyperammonemia, which can lead to ataxia, lethargy, and death without rapid intervention [3]. The estimated frequency of OTC deficiency is 1/50,000–80,000. The estimated frequency of urea cycle disorders is collectively 1/35,000. However, because urea cycle disorders such as OTC deficiency often go unrecognized, these disorders are under-diagnosed, making it difficult to determine the true frequency of urea cycle disorders in the general population [4]. The recommended protein intake for individuals with OTC deficiency at ages between 7 and 19 years are 0.7–1.4 g/kg/day of total protein, 0.4–0.7 g/kg/day of protein from essential amino acid medical food, and 0.3–0.7 g/kg/day of natural proteins [5].

The standard therapy for adults that were diagnosed with OTC deficiency at infancy is directed at reducing the requirement for urea biosynthesis; this is achieved by using a low-protein diet and by increasing waste nitrogen excretion through alternative pathway therapy [6]. However, some stressors such as fasting, a high-protein diet, intercurrent illness, or surgery can trigger episodes of hyperammonemic encephalopathy accompanied by nausea, vomiting, headache, erratic behavior, delirium, aggressive behavior, and lethargy. These episodes can also result in hyperammonemic coma [7,8].

Environmental stressors have been well-established, but the contextual stressors, especially the patient’s personal relation with them, have not. In this line, it has been shown how high-stress situations can produce modifications in the autonomous nervous system, thus increasing the sympathetic branch that modifies the cardiovascular and metabolic response of the subjects [9,10]. In line with this, some authors have found how different eliciting contexts with large psychological stress produce increases in sympathetic modulation, increases in blood glucose and lactate concentrations, increases in muscular strength manifestations, a decrease in operative memory, and cortical arousal [11,12,13,14,15,16]. Autonomic, cardiovascular, and metabolic modifications in a stress context are modulated by the subject’s psychological profile, which would determine their interpretation of the contextual information, their threat perception, and the way they relate with the eliciting context [17,18]. In this way, anxiety profiles have shown an exacerbated response to stressors, showing greater sympathetic activity, which can directly affect both the cardiovascular and metabolic responses [19].

Therefore, considering that OTC deficiency is a metabolism disorder, the effect of stress at the psychological level for this pathology has not been previously described. For this reason, we conducted the present case report in order to analyze the effect of psychological stress on the acute symptoms of a patient with OTC deficiency.

## 2. Case Description

We analyzed an 18-year-old male motorcycle racer who was a participant in the FIM Road Racing World Championship Grand Prix. He was 179 cm tall, with a body composition of 63 kilograms, of which 37.8 were lean muscle mass. He had 6.2% body fat, good fitness (68 mL·kg^−1^·min^−1^ VO_2_max), no injuries, and perfect health. He was without any previously reported physiopathologies and had been diagnosed with Ornithine Transcarbamylase deficiency. The OTC was diagnosed during the infancy period, when the participant presented some symptomatology and was hospitalized because of it. The week prior to the sporting event, the athlete performed high-intensity physical conditioning work in a hot environment (35 °C), with the aim of acclimatizing to the hot environment of the circuit (38 °C), which was where the incident occurred. During this training period prior to competition, cortisol, blood lactate, urine protein, nitrate and glucose levels, and autonomic modulation were controlled, without any alarming results.

The episode analyzed in the present research occurred during a World Championship Grand Prix; this was located in his hometown, where the pilot could have the points necessary to reach the first position of the championship. These situations made this grand prix a highly exciting and stressful context for the driver due to the consequences that its result could bring to his professional career. 

On the first day of competition, during free practice, and after the first round, the athlete returned to the paddock, reporting nausea and dizziness while driving his motorcycle. Once he stopped at the box, his symptoms got worse and he became unconscious. He was rapidly attended to by the medical personnel of the circuit, and once stabilized, he was taken to the local hospital for further analysis and monitoring. He had a history of urea cycle disorder because of OTC deficiency, although he was also following the treatment for the disorders regularly and correctly. He had no alcohol or drugs habits, not even occasionally. He had not been exposed to hepatotoxic chemicals or other substances. Indeed, the subject had not consumed any type of supplement or ergogenic aid of any kind. A blood analysis on hospital admission showed hyperammonemia (307 μg/dL) and excess alkalosis. Nevertheless, other data from the blood and urine analyses, including blood count, liver and renal function tests, blood glucose, electrolytes, and coagulopathy, showed normal range values (Table 1). Regarding arterial gasometry, the data showed excess alkalosis (Table 1). Furthermore, the excess protein and urobilinogen content in his urine from the results of the analysis was a clear sign of liver disease.

## 3. Discussion

This research aimed to analyze the effect of psychological stress on the acute symptoms of a patient with OTC deficiency. Previous studies conducted in populations exposed to high psychological stress, such as the military population, found that psychological stress can trigger a disproportionate metabolic and autonomic response as compared to the physical and mechanical workload load—a fact that had been proven through large blood lactate concentrations, an elevated heart rate (even higher than the individual, theoretical, maximum heart rate), and sympathetic modulation [10,20,21,22]. Even in the academic context, the exposure to psychological stressors such as clinical evaluations, laboratories, final dissertations, or objectively structured clinical examinations produced a large cardiovascular and sympathetic response [11,23,24,25].

In the present report, we described the case of a patient who suddenly started to feel nausea and dizziness during a motorcycle competition and then fell unconscious after a few minutes due to an episode of hyperammonemia. The patient had previously been diagnosed with OTC deficiency; however, he was correctly following the nutritional treatment he had been prescribed. Furthermore, the only stressors that the rider experienced during the grand prix was the race itself (where a real-life risk existed) and his own pressure on himself due to the consequences of the final classification in that grand prix. No other environmental stressor could have triggered the episode that occurred. Therefore, the hyperammonemia episode could have been caused by the psychological stress that the rider suffered, as well as its somatization due to the risky activity and the pressure from the race that the patient took part in. This situation, where the eliciting environment produces an increased threat perception, activates the phylogenetic survival response modulated by the autonomous nervous system, thus increasing sympathetic modulation in order to prepare the organism to face the threat [26,27]. This psychophysiological response produces an increase in the metabolism of the participants, consequently increasing the metabolic demands of anaerobic and aerobic metabolic systems [28,29].

This increase in metabolic stress induces an increased mitochondrial function that leads to elevated levels of acetyl-coenzyme A [30], a fact that increases carbamoyl-phosphate and bicarbonate production resulting from the mitochondria activity [31]. As OTC deficiency precludes carbamoyl-phosphate giving up its carbamoyl group to ornithine, the accumulation of amino groups is accentuated, therefore increasing the concentration of the urea analyzed in the patient. In this line, the over-production of bicarbonate also promotes the alkalosis status evaluated in the patient, which is what triggered the episode of hyperammonemia.

## 4. Conclusions

To our knowledge, no case report of hyperammonemia without a trigger, other than a wrong diet out of treatment or a physically traumatic experience, has been described. Thus, the cascade of physiological events that produced the hyperammonemia of the patient could have been due to the somatization of the psychological stress produced by the eliciting context of the grand prix. Consequently, this case highlights the necessity for the psychological control and evaluation of patients with OTC deficiency, since psychological stress could trigger a metabolism disorder.

## Figures and Tables

**Table 1:** Blood and urine analyses on hospital admission.

	Results	Reference Values
Hematology		
Red blood cells (×10^6^/µL)	4.92	4.30–5.75
Hemoglobin (g/dL)	13.8	13.5–17.2
Hematocrit (%)	42.9	39.5–50.5
Mean corpuscular volume (fL)	87.2	80.0–101.0
Mean corpuscular hemoglobin (pg)	28.0	27.0–34.00
Mean corpuscular hemoglobin concentration (g/dL)	32.2	31.5–36.0
Red blood cell scattering (%)	12.8	11.6–14.5
Erythroblasts (×10^3^/µL)	0	0–4
Leukocytes (×10^3^/µL)	6.39	4.00–11.00
Neutrophils (%)	52.80	42.00–77.00
Lymphocytes (%)	39.10	20.00–44.00
Monocytes (%)	7.00	2.00–10.00
Eosinophils (%)	0.50	0.50–5.50
Basophils (%)	0.60	0.50–1.00
Neutrophils (×10^3^/µL)	3.37	1.50–7.70
Lymphocytes (×10^3^/µL)	2.50	0.90–5.00
Monocytes (×10^3^/µL)	0.45	0.10–0.90
Eosinophils (×10^3^/µL)	0.03	0.02–0.55
Basophils (×10^3^/µL)	0.04	0.00–0.20
Platelets (×10^3^/µL)	175	130–400
Mean platelet volume (fL)	11.8	5.9–15.0
**Biochemistry**		
Glucose (mg/dL)	110 *	70–105
Creatinine (mg/dL)	0.90	0.72–1.25
Gamma glutamyl transferase (U/L)	11	2–42
Lactate dehydrogenase (U/L)	220	125–220
Sodium (mEq/L)	144	136–145
Potassium (mEq/L)	3.8	3.5–5.1
Ammonium (µg/dL)	307 *	31–123
C-reactive protein (mg/dL)	<0.4	0.0–5.0
**Gasometry**		
Ionic calcium (mg/dL)	4.64	4.60–5.10
pH	7.45 *	7.32–7.43
Partial pressure of CO_2_ (mmHg)	43.0	41.0–54.0
Partial pressure of O_2_ (mmHg)	85.0 *	35.0–44.0
O_2_ saturation (%)	96.6 *	65.0–78.0
Total content of CO_2_ (mmol/L)	31.2 *	22.0–26.0
Current bicarbonate (mmol/L)	29.9 *	24.0–28.0
Standard bicarbonate (mmol/L)	28.9 *	21.8–26.2
Excess bases in blood (mmol/L)	5.2 *	−2.0–3.0
Oxyhemoglobin (%)	95.9 *	40.0–70.0
Deoxyhemoglobin (%)	3.4 *	25.0–55.0
Carboxyhemoglobin (%)	0.6	0.5–1.5
Methemoglobin (%)	0.1 *	0.4–1.5
**Urine analysis**		
Urine specific gravity	1.025	1.005–1.025
Creatinine (mg/dL)	7.5	5–8
Proteins (mg/dL)	20 *	0–14
Glucose (mmol/L)	0.00	0.00–0.08
Bilirubin (mg/dL)	0.00	0.00–1.20
Urobilinogen (mg/dL)	3.00 *	0.00–1.00

* values out of normal range.

## Data Availability

Not applicable.
